# Epithelial–Mesenchymal Transitions Leading to Conceptus Adhesion in Ruminants: Early Pregnancy Events in Cattle

**DOI:** 10.3390/ijms26083772

**Published:** 2025-04-16

**Authors:** Mohamed Samy Yousef, Kazuhiko Imakawa

**Affiliations:** 1 Research Institute of Agriculture, Tokai University, Kumamoto 862-8652, Japan; 2 Department of Theriogenology, Faculty of Veterinary Medicine, Assiut University, Assiut 71515, Egypt

**Keywords:** trophoblast, endometrium, conceptus, elongation, epithelial–mesenchymal transition, pregnancy, ruminant

## Abstract

Trophoblast–endometrium interactions play a critical role in the processes of conceptus elongation, attachment, and adhesion, followed by placental development during early pregnancy in ruminants. The attachment between uterine epithelium and trophoblast cells, which is epithelial in nature, requires epithelial to mesenchymal transition (EMT), where the fetal trophoblasts come into contact with maternal epithelial cells without fully invading the maternal tissues. Understanding the early developmental period driving EMT processes in utero in ruminants is fundamental to improving fertility through the prevention of early pregnancy failure and enhancing overall reproductive efficiency in livestock. This review highlights the key events necessary for the early conceptus to progress properly towards firm adhesion with the endometrium, focusing on trophoblast–endometrium interactions. This field holds the potential to elucidate molecular and cellular mechanisms associated with trophoblast and endometrium attachment and adhesion, leading to reduced early embryonic losses and enhanced economic sustainability by developing effective reproductive management strategies.

## 1. Introduction

Enhancing reproductive efficiency in livestock through reducing pregnancy loss will decrease the number of animals needed to meet milk and meat production demands. Culling non-productive or infertile animals reduces methane and nitrous oxide emissions and may contribute to addressing the global warming issue [1]. To date, embryonic death is a significant issue in cattle reproduction, leading to considerable economic losses in livestock industries. Early embryonic mortality in dairy cows exceeds 50% of total embryonic loss [2,3], which includes pregnancy losses occurring between conception and conceptus attachment to the endometrium [4,5]. This phase involves trophoblast differentiation, maternal recognition of pregnancy, and conceptus attachment and adhesion to the endometrial epithelium [6]. Despite various efforts and management interventions during this period, this loss has shown no improvement to date. Embryo implantation in all mammals requires a series of pivotal events to ensure successful progression of pregnancy, including uterine receptivity, the attachment between the trophoblast and endometrial epithelium, and the interplay between trophoblast cells and the uterus [7]. In particular, the attachment between the elongated conceptus and the endometrium in ruminants involves bidirectional signaling, facilitated by epithelial–mesenchymal transition (EMT), adhesion molecules, hormones, and cytokines that promote trophoblast adhesiveness and endometrial receptivity [8,9]. Any disturbance in these processes can result in embryonic death [10,11,12]. In addition, the intrinsic embryonic factors can significantly influence the development and viability of ruminant embryos. For instance, embryonic gene activation and proper cell differentiation, as well as genetic defects including homozygous recessive genes, can arise from inbreeding [13,14,15], and chromosomal abnormalities such as mixoploidy lead to reduced embryo viability and pregnancy failure in bovines [16].

Despite species-specific differences in placental structures, all mammalian placentas have trophoblasts that interact intimately with the uterine endometrium to control and guarantee the transfer of nutrients from the mother to the embryo/fetus via coordinated microcirculatory systems [17,18,19]. Studying the events during the peri-implantation period in ruminants is challenging due to the difficulty of replicating maternal-fetal interactions in vitro, and most research regarding this period is directed toward human and mouse models [20,21,22]. This review explores the current understanding of the developmental events related to the conceptus attachment to the maternal endometrium, highlighting lessons learned from human and mouse models, and underscores the need for advanced models to better understand crosstalk between the early conceptus and uterine endometrium during this period in ruminants. Even a minor disturbance in the intricate system during conceptus elongation and the attachment process in utero can lead to pregnancy failure. This review provides a thorough overview of current research that can result in the development of diagnostic and therapeutic strategies for early pregnancy disorders, such as early embryonic death and idiopathic infertility.

## 2. Trophoblast–Endometrium Interaction

Ruminant placentas exhibit unique characteristics compared to those of other mammals. They are classified as cotyledonary and synepitheliochorial, respectively, depending on their gross anatomy and histological features [17,18,23]. The process of placental formation starts before the attachment of the early elongated conceptus to the maternal endometrial epithelium. Therefore, the early embryonic development, trophoblast differentiation, and migration are crucial for proper implantation [24]. Placentation in ruminants initially develops as an epitheliochorial (non-invasive) placenta and then transforms into a synepitheliochorial (semi-invasive) type during the early pregnancy period [17]. However, the ruminant placenta was misclassified first as syndesmochorial due to difficulties in distinguishing the layers separating the maternal and fetal circulations [25]. In the meantime, the earlier presumption was that the uterine epithelium is lost, leading to direct contact between the trophectoderm and the maternal connective tissue. It is now understood that the uterine epithelium (UE) is not lost but modified by fusion with the migrated trophoblasts (binucleate cells, BNCs), forming the fetomaternal syncytium [25,26]. A recent immunohistochemical study suggests a more complex scenario at the bovine uteroplacental interface [27]. It proposes that some PAG-positive cells are mononuclear, with possible migratory abilities. The same study found that by day 21 of gestation in cows, certain regions of the luminal epithelium (LE) were disrupted by the presence of PAG-positive trophoblast giant cells (TGCs). By day 31, large sections of the LE were missing, and by day 40, the LE was completely absent at placental attachment sites and replaced by a thick layer of PAG-positive cells (syncytia). These findings indicate that the bovine placenta may function as a syndesmochorial type at least part of pregnancy [27]. In fact, during conceptus elongation and its early attachment to the endometrium, BNCs differentiate from uninucleate cells (UNCs) in the trophectoderm (TE) of the chorion [8,11] through endoreduplication (mitosis without cytokinesis) [28,29].

In bovines, BNCs migrate and alter the uterine epithelium by apical fusion to form hybrid syncytial plaques, with up to eight nuclei at the interface between the fetal and maternal tissues [26,30]. By day 40° (day 0 = day of estrus), these plaques are replaced by the regrowth of UE cells and the subsequent fusion of trophoblast giant cells (TGC) with the UE, resulting in the formation of transient trinucleate minisyncytia [30]. It should be noted that BNCs do not proliferate, and the trophoblast cells do not penetrate the basal lamina of the LE [8,17]. BNCs represent about 15–20% of the chorion trophectoderm. Moreover, BNCs are rich in secretory granules in their cytoplasm that constitute about 50% of the total cell volume. Further, the secretory granules can leave the trinucleate cells to the endometrial stroma and transfer the proteins to the maternal circulation [29]. This potentially acts as a bridge for immunomodulatory and metabolic signals between the fetal and maternal circulations [30]. In the same way, intraluminal vesicles of ruminant BNC granules are considered a possible source of placental exosomes to support fetomaternal communication [31]. In ruminants, historically, BNCs were thought to undergo heterotypic (trophoblast–uterine epithelial cell) fusion to form trinucleated cells, based on anatomical and morphological evidence [32]. However, recent studies from our research group and others [33,34] suggest that the multinucleated cells observed in ruminants mostly arise from homotypic (trophoblast–trophoblast) fusion. Notably, single-cell analysis of bovine placenta revealed two types of UNCs and three different populations of BNCs [17]. These findings suggest an additional cellular mechanism (homotypic fusion) that may have favored the evolution and reproductive adaptations in ruminants by minimizing the direct interaction between the maternal and fetal sides.

## 3. Conceptus Elongation and Attachment to Endometrium

**Figure 1:**
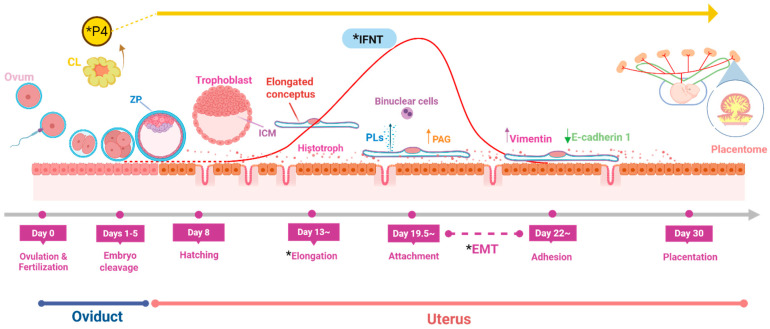
Developmental timeline of the bovine embryo from fertilization to uterine adhesion. Ovulation happens on day 0, followed by fertilization in the oviduct. Then, the embryo undergoes early cleavage and development in the oviduct till days 4−5. By days 5−6, the blastocyst moves to the uterus, where it hatches from the zona pellucida, a process potentially associated with increased secretion of interferon tau (IFNT). IFNT secretion begins as early as day 4 in the oviduct, reaching peak levels between days 14−20. The conceptus begins to elongate on days 14−15, as it transforms from ovoid form to a filamentous structure, and binucleate cells (BNCs) appear from the trophoblast. Around day 19.5, it starts to attach to the uterine epithelium. BNCs secrete placental lactogens (PLs) and pregnancy-associated glycoproteins (PAGs); at this stage, epithelial–mesenchymal transition (EMT) initiates. Then, 2 days later (~day 22), the conceptus becomes firmly adhered to the uterine epithelium, and the molecular markers of EMT, such as a decrease in E-cadherin 1 (epithelial marker) and an increase in vimentin (mesenchymal marker), are detected. * Key critical points that can compromise early pregnancy in ruminants include insufficient production of progesterone (P4) or interferon tau (IFNT), as well as delayed or failed conceptus elongation and epithelial–mesenchymal transition (EMT), which can impair embryonic development and the implantation process. This figure was created with BioRender. Available from: https://BioRender.com/a778kz3 accessed 12 April 2025.

In ruminants, the blastocyst does not implant in a spherical form. Before implantation, the blastocyst undergoes growth and elongation processes for about two weeks after entering the uterus [35]. In cattle, the conceptus elongation begins on days 13–14 and progresses for attachment to the UE on days 19–20 (Figure 1) [36]. During this period, the gene expression profiles of the conceptus alter dramatically with the increased activity of genes responsible for cell growth and metabolism [37] and high production of interferon-tau (IFNT), the pregnancy recognition signal in ruminants. Conceptus elongation is characterized by dramatic increases in trophectoderm length and weight, primarily because of the rapid proliferation of trophectoderm cells rather than changes in cell shape. The conceptus is elongated in cattle from an ovoid shape within days 12 to 14, approximately measuring 2 mm on day 13, into 60 mm by day 16 and reaching about 250 mm on day 19 [38,39]. In fact, the trophoblast length doubles daily from days 9 to 16 after hatching, with a sharp increase between days 13 and 14, leading to a more than 1000-fold increase in size by day 19, when IFNT production reaches the peak [38,39,40,41].

Of particular note, the interaction between the early conceptus and the uterine environment is crucial, as the conceptus relies on endometrial–luminal nutrients for elongation [39,42] and cannot elongate under the existing in vitro culture system [43,44]. The required nutrients and signals for the conceptus elongation are provided by the uterine histotroph, which is mainly secreted by uterine glands, and modulated by ovarian steroids and conceptus-derived molecules [11]. The histotroph is a complex mixture of proteins, lipids, amino acids, sugars, ions, and microvesicles [45]. Ewes with uterine gland knockout display infertility and exhibit recurrent early pregnancy loss [46]. Thus, conceptus elongation is a maternally dependent process [45], as it does not happen either in vitro or in vivo in the absence of uterine glands [46].

Strikingly, epithelial–mesenchymal transition (EMT) is a crucial process during this period, enabling trophectoderm cells to acquire migratory and invasive properties. EMT plays a pivotal role in physiological events like embryonic development and pathological conditions such as tumor formation and metastasis [47]. EMT can be categorized into three distinct types based on the biological context:

Type (1) enables the transient transformation of epithelial cells into mesenchymal cells, contributing to the formation of mesoderm and endoderm. This type is associated with embryo formation, implantation, and organ development and does not result in fibrosis or excessive metastasis (e.g., systemic invasion). Type (2) is associated with wound healing, tissue regeneration, and organ fibrosis [48], while Type (3) is linked to cancer that promotes clonal outgrowth and metastasis [49]. In ruminants, the differentiation of mononuclear trophoblast cells into BNCs, which acquire migratory and invasive properties, occurs via Type (1) EMT prior to conceptus attachment (days 19–20) [36]. Further, conceptus trophectoderm expresses EMT markers on day 22 and starts the firm adhesion to the maternal side [36]. This transition plays a critical role in trophectoderm attachment to the uterine epithelium, promoting conceptus implantation.

**Figure 2:**
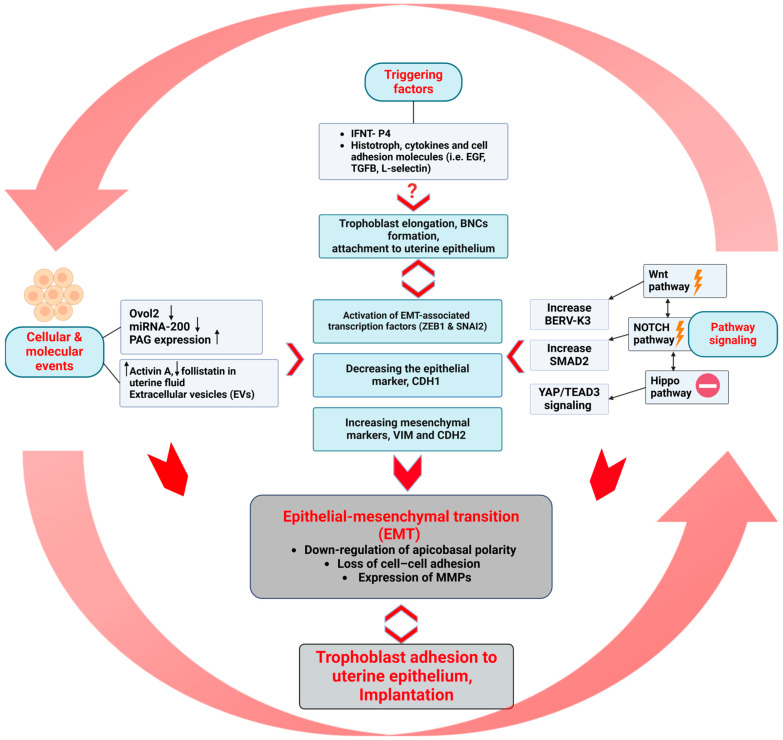
Key events of epithelial–mesenchymal transition (EMT) prior to implantation in the bovine. EMT is triggered by factors including interferon tau (IFNT), secreted from the very early embryo (~day 4) with peak levels at days 14–20, progesterone (P4), uterine histotroph enriched with cytokines, growth factors (e.g., EGF and TGF-β), and adhesive molecules (e.g., L-selectin) along with unidentified factors (?). These support elongation and attachment of the conceptus to the uterine endometrium. EMT involves downregulation of Ovol2 and miRNA-200 [50], while the expression of pregnancy-associated glycoproteins (PAGs) increases [51], and changes in uterine fluid (e.g., increased activin A, decreased follistatin [51]), and extracellular vesicles (EVs) secreted by both the trophoblast and uterus [52]. Key signaling pathways include Wnt (upregulating BERV-K3) [53], NOTCH (activating SMAD2) [54], and Hippo inhibition (YAP/TEAD3 signaling) [50]. These lead to activation of EMT-associated transcription factors (ZEB1 and SNAI2), a decrease in epithelial markers [E-cadherin 1 (CDH1)] [50], and upregulation of mesenchymal markers [Vimentin (VIM) and E-cadherin 2 (CDH2)] [50]. These pathways and molecular changes enable the trophoblast to lose apicobasal polarity, reduce cell–cell adhesion, express matrix metalloproteinases (MMPs), and transition to a mesenchymal state. This facilitates trophoblast adhesion, leading to successful implantation in ruminants.

Based on our previous studies and those of others, the key features of EMT in ruminant trophoectoderm (Figure 2) can be summarized as follows:

### 3.1. Triggering Agents

IFNT is highly produced by mononucleate trophectoderms between days 14–21 of pregnancy in cows. Along with systemic progesterone (P4), trophoblast IFNT itself or together with blastocysts regulate endometrial gene expression [55,56], resulting in the establishment of a proper uterine lumen environment. P4 and IFNT indirectly support successful trophoblast elongation and promote blastocyst attachment to the uterine epithelium [55]. For instance, IFNT induces the secretion of PGE2 from bovine endometrial epithelial cells, which in turn activates cyclic AMP (cAMP) signaling and increases IGF-binding proteins (IGFBP) and VEGF expression in stromal cells [57]. These could be associated with the functions of cell proliferation, migration, and attachment in ruminants. Moreover, both P4 and IFNT stimulated expression of galectin 15 (LGALS15), cystatin C (CST3), and cathepsin L (CTSL) in the ovine endometrial epithelium. These molecules are known to directly modulate components of the extracellular matrix (ECM); for instance, CTSL degrades ECM proteins [58], while CST3 regulates protease activity [59], and LGALS15 influences growth and cell adhesion [60]. Together, these changes may contribute to the remodeling of the endometrial ECM, facilitating conceptus attachment by modifying the adhesion interface between the endometrial epithelium and the conceptus [56]. Furthermore, IFNT isoforms, such as IFNT2 and IFNTc1, regulate bovine endometrial epithelia through forkhead box S1 (FOXS1) transcription factor [61]. This study proposed the significance of these isoforms in inducing immunoproteasome formation and class I antigen presentation in endometrial epithelial cells, which are required for conceptus–endometrium interaction.

Importantly, we investigated cytokines and cell adhesion molecules such as epidermal growth factor, basic fibroblast growth factor, transforming growth factor beta (TGF-β), activin A, L-selectin-podocalyxin, and vascular cell adhesion molecule 1-integrin α4 expression in the uterus, which contributed to the initiation of EMT in the trophectoderm using an in vitro coculture system with bovine trophoblast CT-1 and an endometrial epithelial cell [55,62]. In our previous study, we collected uterine fluid (UF) from cows on days 17 (P17, pre-attachment) and 20 (P20, in-between attachment), and their protein contents were analyzed. Interestingly, iTRAQ analysis of P17 or P20 UF detected 20,063 proteins, from which 240 proteins were identified as secretory proteins [51]. Among the secretory proteins, numerous proteins belong to the TGF-β family. Additionally, we previously identified conceptus secretory proteins, such as fibrin, on day 21 of pregnancy in ewes, which are likely involved in regulating conceptus attachment and adhesion in ruminants [63].

### 3.2. Cellular and Molecular Events

We found that ovine conceptuses expressed both mesenchymal [vimentin (VIM) and N-cadherin 2 (CDH2)] and epithelial markers [cytokeratin and E-cadherin1 (CDH1)] on day 21 [34]. In the same way, both VIM and CDH2 were expressed in bovine conceptuses along with cytokeratin on day 22 [64]. Such epithelial–mesenchymal (E/M) intermediate states may be essential for the acquisition of adhesive properties while limiting the invasive ability of the trophoblast cells [65]. Further, OVO-like zinc finger 2 (OVOl2) is considered a pivotal gatekeeper of epithelial identity by maintaining cell-to-cell junctions, polarity, and stationary behavior. We previously reported that the downregulation of OVOl2 induces EMT in the ruminant trophectoderm by activating the EMT-associated transcription factors (zinc finger E-box binding homeobox 1 (ZEB1) and snail family transcriptional repressor 2 (SNAI2), decreasing the epithelial marker, CDH1, and increasing mesenchymal markers, VIM and CDH2 [50]. Additionally, an elevated concentration of activin A along with a lower concentration of follistatin in uterine fluid on day 22 induces the expression of EMT markers in bovine trophoblasts [51]. In addition, the downregulation of miRNA-200 in bovine trophoblast is associated with EMT progression [50].

Further, we found that pregnancy-associated glycoproteins (PAG) transcripts were highly expressed in bovine conceptuses on day 21 compared to the minute expression on conceptus on days 15 and 17. These findings suggest that PAG expression appeared before the onset of EMT [51]. Notably, specific PAGs have been implicated in EMT by promoting cell migration and invasion in cancer [66,67]. However, the exact role of PAGs in regulating EMT in ruminant placentation remains to be fully elucidated. Importantly, extracellular vesicles (EVs) existing in the uterine lumen can facilitate conceptus–endometrial communication prior to implantation by transferring bioactive molecules (i.e., IFNT) that regulate gene expression, cell proliferation, and adhesion, as previously reviewed by Nakamura et al. [52]. Interestingly, trophoblast vesicles appear to have the ability to store IFNT and keep the morphological features of the original bovine trophoblasts [68]. In addition, EVs with micro RNAs (miRNAs) and long non-coding RNAs (lncRNAs) have been found to play a crucial role in the establishment of the proper uterine environment required for the peri-implantation processes [69,70,71].

### 3.3. Pathway Signaling

The spatiotemporal expression of canonical wingless/T-cell factor signaling pathway (Wnt) components highlights the dynamic regulation of Wnt signaling in bovine embryonic development and embryo attachment [72]. Wnt receptor Frizzled-4 (Fzd4) and β-catenin, transcriptional regulator of Wnt signaling, were identified in bovine BNCs, but not in UNCs [72]. Dickkopf-1 (DKK1) is an endometrial secretory protein, which inhibits the Wnt pathway by disrupting the formation of the Wnt ligand/Fzd complex, promoting embryonic differentiation in bovine and humans [72]. Quite recently, DKK1 was applied in vitro to enhance blastocyst development and decrease apoptosis in bovine embryos [73]. On the other hand, our previous studies demonstrated a decrease in DKK-1 expression in day 22 bovine conceptuses [74]. Of particular note, Wnt agonist induces endogenous BERV-K3 expression, enhancing fusion properties in bovine trophoblast [53], and increases cell migration in ovine trophectoderm [75]. Inhibiting the Wnt pathway can reverse EMT in cancer cells by decreasing the expression of EMT-inducing transcription factors (SNAI2 and twist homolog 1(TWIST)) [76].

Further, the activation of the Hippo pathway leads to inactivation of yes-associated protein (YAP) and prevents the transcription of genes involved in cell proliferation and survival [77]. In contrast, when the Hippo pathway is inhibited, YAP translocates to the nucleus and activates Tetraethylammonium (TEAD; domain transcription factor), which promotes EMT and cell invasion [50]. In addition, TEAD relocation and/or YAP degradation following its phosphorylation downregulates IFNT gene transcription after bovine conceptus attachment to the uterine endometrium [78]. Hippo signaling regulates the downregulation of OVOl2 during bovine conceptus implantation into the uterine endometrium and enables EMT progression on day 22 [50].

We previously stated that functional silencing of NOTCH2 promotes EMT in bovine trophoblast cells [50]. In this study, we showed the potential crosstalk between NOTCH signaling with OVOl2 and Hippo signaling to regulate the EMT process via upregulation of SMAD2, SMAD3, and/or SMAD4 in bovine conceptuses. Similarly, NOTCH signaling also interacts with other cell signaling pathways, such as Wnt, intensifying the invasiveness of tumor cells [54]. These findings reveal the potential significance of the interactions between Wnt, Hippo, and NOTCH signaling pathways to initiate and promote EMT in the ruminant trophectoderm in a regulated manner to avoid excessive invasion.

## 4. Insights from Mouse and Human Models

Extensive studies have been conducted to elucidate the mechanisms responsible for trophoblast differentiation and immune modulation during placental development using mouse and human models. Keeping in mind the species-specific placental biology, many molecular processes are conserved across species. The main differences between these placentas can be briefly summarized as follows: (1) Shape and structure of the placenta; ruminants have synepitheliochorial–noninvasive placenta, while, in humans and mice, it is discoidal and hemochorial-invasive. (2) Histotroph (uterine secretions); it is essential for embryonic development and serves a critical function during the pre- and early implantation phases. Early conceptus in ruminants depends heavily on the histotroph for days during early development before implantation. Spherical blastocysts in humans and mice implant directly into the endometrium and do not remain free in the uterus for an extended period before attachment. (3) Maternal recognition of pregnancy (MRP): ruminant conceptuses produce IFNT from the peri-implantation trophectoderm; such mechanism is absent in humans and mice. Despite such differences, the extracted insights from these models may help to decode the complexity and unknown mechanisms in ruminant placentation.

### 4.1. Immune Modulation at the Maternal-Fetal Interface

The major histocompatibility complex (MHC) expression in ruminant early placentas is limited compared to the hemochorial placentation seen in humans and mice to avoid immune rejection of the conceptus. Classical MHCs are almost absent on trophoblast cells of ruminants [79,80]. During the MRP, IFNT stimulates MHC class I expression in the endometrial stroma, but not in the trophoblasts of ovine conceptuses [79]. In cattle, the upregulation of MHC class I on fetal (binucleate) trophoblasts induces a maternal-fetal alloimmune response that is essential for the required loss of maternal-fetal adherence [81]. By contrast, human and mouse trophoblast cells express both classical and nonclassical MHC to modulate maternal-fetal immune interactions in such invasive states [82,83,84,85]. Although IFNT regulates the uterine environment [55,56] and allows blastocyst attachment to the uterine epithelium [55], immune responses to the early conceptus are not exactly the same between the first pregnant heifers and multiparous cows [86].

In the same way, the distribution and numbers of immune cells are different among the embryo–maternal interface of those species. For instance, natural killer (NK) cells are less abundant at the maternal-fetal interface in ruminants compared to the large numbers existing in human and mouse placentas [87]. In ruminants, IFNT induces CXCL10 and CXCL11 in endometrial tissues [88,89], which in turn attracts immune cells such as NK cells to the caruncular regions [90]. In addition, through the CXCL10 receptor, CXCR3, this chemokine regulates trophectodermal cell migration and integrin (ITGs) expression [88]. Cell–cell interaction between NK cells and trophoblast cells in humans is well defined, involving the HLA-G cycle, Gal-9/Tim-3, KIR-HLA, and LILRB1-HLA-G signals, as previously reported by Liu et al. [91]. The precise role of NK cells in ruminant placentation remains undefined.

### 4.2. Retrovirus Gene Expression in Placenta

The notion of involving endogenous retroviral (ERV) genes in early placentation and evolution was first recognized in humans [92] and mice [93] after the identification of Syncytin-1 and proof of its roles in trophoblast fusion. Later, Syncytin-Rum1 was identified across ruminants and was absent in non-ruminants [94]. Additionally, other ERV genes (i.e., BERV-K1, -K2, -K3, and -P) are expressed in the bovine placenta [53,94,95]. Quite recently, BERV-K1 was identified in the extracellular vesicles released from the bovine fetal binucleate trophoblast cells, suggesting its role in maternal-fetal communication [96]. Wnt agonists induce the expression of bovine endogenous retroviruses (BERV-K3) in bovine trophoblast cell lines [53]. ERV genes were proposed as an evolutionary model for understanding placental function and adaptation in ruminants [55]. Syncytin-Rum1 was integrated into the bovine genome over 30 million years ago, before the integration of BERV-K1, which appeared approximately 18.3 to 25.4 million years ago [97,98,99]. However, BERV-K1 shows higher fusogenic activity compared to Syncytin-Rum1 [99]. Of note, ruminants, with their unique synepitheliochorial placenta, show the highest evolutionary success after K/Pg (200+ species), surpassing odd-toed ungulates (~9), pigs (~30), and aquatic mammals (~90) [30]. For more information related to ERVs and their integration into the mammalian genomes and placental diversion, see Imakawa et al., [95]. However, the functional significance of some ERV genes is still unclear. While ERVs are often investigated for their beneficial role in placentation, it is essential to consider whether some ERVs have an adverse effect on mammals’ placentation.

### 4.3. Hypoxia and Oxygen-Sensing

Hypoxia-inducible factors (HIFs) are essential for trophoblast differentiation and early placental development in mice and humans [100,101,102]. However, prolonged or extreme hypoxia, such as in high-altitude conditions or maternal illness, resulted in placental insufficiency (e.g., intrauterine growth restriction, IUGR) [103]. Notably, abnormal oxygen sensing leads to defective vascularization and impaired trophoblast invasion in the hemochorial placenta [102] and inhibit P4 secretion in the synepitheliochorial placenta [104]. Adaptive mechanisms, including characteristic trophoblast invasion, angiogenesis, and other cellular processes (e.g., increased glycolysis and regulation of mitochondrial function), might be crucial for optimizing oxygen tension and HIF signaling in the ruminant placenta under physiological conditions, due to the structure feature, or stress factors, such as grazing at high altitudes. Vascular endothelial growth factor (VEGF) is a potential candidate to adapt to the challenge posed by the non-invasive placental structure in ruminants. VEGF is a key regulator of placental angiogenesis, promoting endothelial cell proliferation, migration, and survival in mice, humans, and ruminants [105,106,107]. The VEGF pathway is used in ruminant placentation to enhance vascular branching within cotyledons and compensate for the lack of direct maternal vascular contact [108,109].

### 4.4. Circulating Placental RNAs to Predict Pregnancy Complications

Circulating RNA of feto-placental origin was first detected by Lo et al. in 1997 through the detection of Y chromosome-specific RNA in the blood of women pregnant with a male fetus [110]. Measuring placental RNAs in maternal circulation is now proposed as a non-invasive biomarker to predict pregnancy complications and monitor placental status [111]. To date, there is a lack of well-identified diagnostic criteria for placental health in ruminants throughout the gestation period. Circulating placental RNA may offer a diagnostic method for assessing placental health, leading to management strategies and subsequently improving fertility outcomes in livestock. In ruminants, IFNT cannot be detected outside the uterus. This may be due to limited diffusion through the uterine tissue or rapid degradation in the blood circulation. However, the antiviral activity of IFN increases in the uterine vein during early pregnancy in sheep, as previously reported by Romero et al. [112], and the interferon-stimulated gene expression is upregulated in peripheral immune cells and different organs of cows and sheep [113,114]. Imakawa et al. [69] proposed a novel mechanism by which IFNT is carried by uterine EVs, originating from both conceptuses and the uterine endometrium, entering the bloodstream and being responsible for the systemic effect of IFNT. Successful isolation and detection of such EVs in the blood during early pregnancy in ruminants can be an alternative method for early pregnancy diagnosis.

## 5. Research Challenge

Blastocysts can be successfully produced in vitro, but with significantly lower rates and quality compared to those produced in vivo. Notably, traditional in vitro and ex vivo models are not capable of supporting the elongation of the conceptus or the full development of placentation until term [115]. In vitro-cultured bovine trophoblastic cells (BT-1) can differentiate into BNCs expressing placental lactogen, mimicking the in vivo characteristics [116,117]. However, the migratory properties of these BNCs and their interactions with the maternal side remain uncertain.

Previously, we developed an in vitro coculture model using trophoblast cells and uterine epithelial cells to investigate the factors involved in the attachment of trophoblast cells to the uterine epithelium after exposure to uterine flushing [62]. However, mimicking the complicated uterine environment at the time of attachment of trophoblast cells is challenging due to the numerous factors involved, such as hormones, cytokines, growth factors, the histotroph, and unidentified factors, along with their interactions throughout the elongation and attachment processes [118]. New protocols utilizing bovine organoids, microfluidics, and bioinformatics approaches for better understanding the complex signaling between the conceptus and uterine tissue during this critical period have been recently proposed [119]. Developing a representative model that mimics the biological system for embryo–maternal crosstalk is essential for comprehending the complex events involved in early pregnancy establishment. Such a model could offer valuable insights and promote the research approach for innovative strategies to enhance fertility in ruminants.

## 6. Conclusions

Overall, the current review demonstrates the complexity and coordination of various cellular and biochemical factors essential for the key events during the pre-implantation period in ruminants. A deeper understanding of these events should aid in the development of novel strategies to mitigate early pregnancy losses and enhance reproductive management in livestock. Advanced technologies such as single-cell sequencing, gene-editing tools, and organoid culture models—along with the insights from human and mouse placentas—offer promising opportunities to untangle the complexities of conceptus—endometrium interactions in ruminants.
